# ABCD3-I and ABCD2 Scores in a TIA Population with Low Stroke Risk

**DOI:** 10.1155/2021/8845898

**Published:** 2021-02-25

**Authors:** Fredrik Ildstad, Hanne Ellekjær, Torgeir Wethal, Stian Lydersen, Hild Fjærtoft, Bent Indredavik

**Affiliations:** ^1^Department of Neuromedicine and Movement Science, Faculty of Medicine and Health Sciences, Norwegian University of Science and Technology (NTNU), Trondheim, Norway; ^2^Department of Medicine, Stroke Unit, Trondheim University Hospital, P.O. Box 3250, N-7006 Trondheim, Norway; ^3^Regional Center for Child and Youth Mental Health and Child Welfare, NTNU, P.O. Box 8905, N-7491 Trondheim, Norway; ^4^Department of Medical Quality Registries, Trondheim University Hospital, P.O. Box 3250, N-7006 Trondheim, Norway

## Abstract

**Objectives:**

We aimed to evaluate the ABCD3-I score and compare it with the ABCD2 score in short- (1 week) and long-term (3 months; 1 year) stroke risk prediction in our post-TIA stroke risk study, MIDNOR TIA.

**Materials and Methods:**

We performed a prospective, multicenter study in Central Norway from 2012 to 2015, enrolling 577 patients with TIA. In a subset of patients with complete data for both scores (*n* = 305), we calculated the AUC statistics of the ABCD3-I score and compared this with the ABCD2 score. A telephone follow-up and registry data were used for assessing stroke occurrence.

**Results:**

Within 1 week, 3 months, and 1 year, 1.0% (*n* = 3), 3.3% (*n* = 10), and 5.2% (*n* = 16) experienced a stroke, respectively. The AUCs for the ABCD3-I score were 0.72 (95% CI, 0.54 to 0.89) at 1 week, 0.66 (95% CI, 0.53 to 0.80) at 3 months, and 0.68 (0.95% CI, 0.56 to 0.79) at 1 year. The corresponding AUCs for the ABCD2 score were 0.55 (95% CI, 0.24 to 0.86), 0.55 (95% CI, 0.42 to 0.68), and 0.63 (95% CI, 0.50 to 0.76).

**Conclusions:**

The ABCD3-I score had limited value in a short-term prediction of subsequent stroke after TIA and did not reliably discriminate between low- and high-risk patients in a long-term follow-up. The ABCD2 score did not predict subsequent stroke accurately at any time point. Since there is a generally lower stroke risk after TIA during the last years, the benefit of these clinical risk scores and their role in TIA management seems limited. *Clinical Trial Registration*. This trial is registered with NCT02038725 (retrospectively registered, January 16, 2014).

## 1. Introduction

Patients with transient ischemic attacks (TIA) are at risk of subsequent strokes, especially early after the attack [1, 2]. Therefore, urgent assessment and intervention is essential in preventing strokes in patients with TIA [3, 4]. Accurate identification of patients at highest risk of stroke after TIA has been considered important in the clinical evaluation and management of these patients. In the last two decades, clinical scores have been established to estimate the stroke risk following a TIA, with the ABCD2 and the ABCD3-I scores being the best validated ones (see Table 1). The ABCD2 score was originally developed to aid nonspecialists in community-based referring settings in management of TIA patients [5]. The ABCD3-I score was developed for use in secondary care and includes information from initial diagnostic investigations [6].

In our prospective TIA study, MIDNOR TIA, we found a lower stroke risk after TIA than reported in earlier studies [7]. The ABCD2 score was able to identify patients with very low risk of stroke, but did not reliably discriminate between low- and high-risk patients, suggesting that it may be less useful in populations with a general low risk of stroke after TIA. The primary aim of the present study was to investigate the predictive accuracy of the ABCD3-I score and secondary to compare it with the ABCD2 score in short- and long-term risk stratification, and to test whether the ABCDI-3 score performed better in populations with a low risk of stroke after TIA.

## 2. Materials and Methods

### 2.1. Study Design and Participants

This is a prospective multicenter study enrolling patients with TIA; the methods of which have been described in detail previously [7]. In brief, all eight hospitals in the region of Central Norway recruited patients from October, 2012, to July, 2014, with a follow-up until July, 2015. Experienced stroke physicians performed inclusion, in most cases on the hospital ward. All patients underwent a standardized diagnostic work-up containing brain and vascular imaging in addition to a detailed patient history, physical examination, blood tests, and cardiac rhythm monitoring. By a telephone follow-up, trained study nurses recorded subsequent stroke (ischemic and hemorrhagic) within 1 week, 3 months, and 1 year after the index TIA. To confirm registered strokes, we used data from the Norwegian Cardiovascular Disease Registry, which includes the Norwegian Stroke Register. All patients were managed according to current treatment guidelines for TIA [8].

Recording of the ABCD2 score was done prospectively as this was the primary aim of the original study, while the ABCD3-I scores were calculated after the study completion by assigning two points for dual TIA, two points for stenosis (≥50%) on carotid imaging, and two points for positive diffusion-weighted imaging MRI (DWI). A positive DWI was defined as ≥1 areas of high signal intensity interpreted as acute ischemic lesions. The abnormal DWI findings were diagnosed by radiologists, in most cases neuroradiologist. Carotid stenosis was defined as a ≥50% narrowing in the lumen of the internal carotid artery that could be responsible for the neurological symptom. The index TIA was defined as the most recent TIA leading the patient to seek medical help. Dual TIA was defined as the occurrence of at least one other TIA during the 7 days before the index event. The blood pressure measurement used for the ABCD2 and ABCD3-I assignment was the first ever recorded after the onset of the TIA.

The TIA diagnosis was based on the World Health Organization criteria [9], which defines a TIA as an acute loss of focal cerebral or ocular function lasting less than 24 hours, without an apparent nonvascular cause. The WHO criteria were also used for stroke [10].

### 2.2. Statistical Analysis

The area (AUC) under the receiver operating characteristic (ROC) for the two scores was estimated using Roger Newson's program—somersd (available in Stata). Somers' D computes the Harrell's C, an equivalent to the AUC, referred to as the AUC here [11]. Perfect prediction produces an AUC of 1.0, whereas prediction that is no better than chance produces an AUC of 0.5. We performed Cox proportional hazards regression analyses to calculate hazard ratios (HRs), using the low-risk ABCD3-I group as the reference category. Cox regression analyses with the covariates positive DWI, dual TIA, and carotid stenosis one at a time were also performed to identify to what degree these additional features in the ABCD3-I score contributed to the predictive value of the score.

Descriptive statistics for continuous variables are given as means with standard deviations (SD) and for categorical variables as frequencies and percentages. Statistical analyses were performed using SPSS Statistics 25 and Stata 15.

## 3. Results

**Figure 1:**
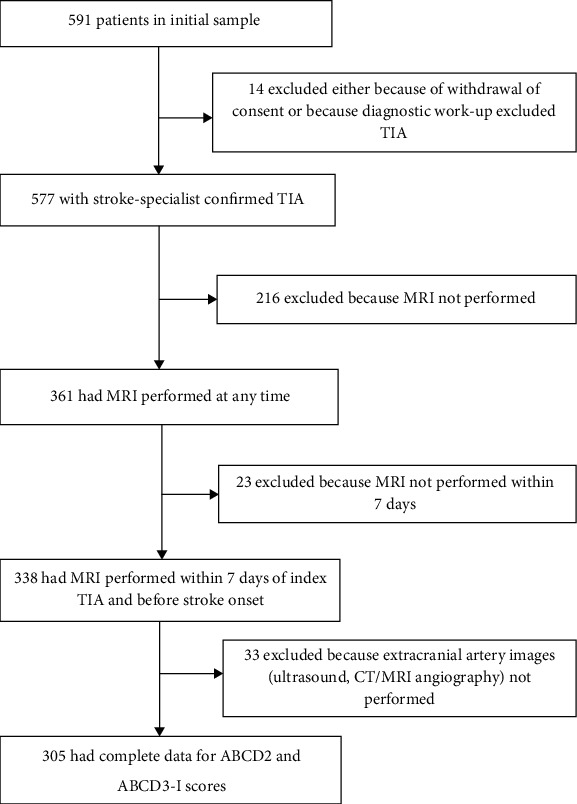
Flow chart of study profile.

Of the 577 patients included in the original study, 305 patients had complete data for secondary analysis of both the ABCD3-I and ABCD2 scores (see Figure 1). The main reason for exclusion was that MRI investigation had not been performed.

Table 2 summarizes the clinical characteristics of the patients included and excluded from the analysis. The mean (SD) age of the included patients was 68.0 years (10.9), of whom 60% were men. Hypertension was the most frequent vascular risk factor. In total, 35 patients (11.5%) had dual TIAs. Ultrasonography was the preferred investigational method of carotid arteries in most cases and was performed in 92% (*n* = 279) of the patients, while CT or MR angiography was performed in 25% (*n* = 75). Twenty-six patients (8.5%) had >50% stenosis of ipsilateral internal carotid artery. Of these, 17 patients (65.4%) underwent carotid surgery (carotid endarterectomy). There were no periprocedural strokes. Acute ischemic lesions on DWI were identified in 89 patients (29.2%). Two hundred and fifty-eight patients (84.6%) were admitted to hospital in less than 24 hours after symptom onset. Eighty-nine (29.2%) had their DWI performed within 24 hours after the index TIA, 63% (*n* = 192) within 48 hours, and 81% (*n* = 247) within 72 hours. Aphasia and dysarthria (46.2%) and arm paresis (34.1%) were the most common symptoms. The number of patients on antiplatelet therapy increased from 37% before TIA to 90.5% (*n* = 276) at the time of discharge from the hospital. Among these, 95% (*n* = 261) were treated with aspirin, either in monotherapy or in combination with dipyridamole (*n* = 133) or clopidogrel (*n* = 20). The patients excluded from the analysis were older and had a higher burden of vascular risk factors, but except for age, former TIA, hypertension, and atrial fibrillation, there were no other significant differences in baseline characteristics between the groups.

Cumulative incidence of stroke was 1.0% (3 patients), 3.3% (10 patients), and 5.2% (16 patients) within 1 week, 3 months, and 1 year after onset of TIA, respectively. Comparing low- and medium- to high-risk ABCD3-I categories, the rate of stroke increased from 0% to 2.5% within 1 week, 0% to 7.5% within 3 months, and 2.1% to 10.0% within 1 year. When comparing low- to high-risk ABCD2 categories, the rate of stroke increased from 0.9% to 1.0% within 1 week, 1.9% to 4.1% within 3 months, and 2.8% to 6.6% and within 1 year (see Table 3).

A Cox regression analysis comparing medium (4–7) and high (8–13) ABCD3-I score with low (reference) score (0-3) showed hazard ratios of 3.84 (95% CI, 0.49 to 30.0; *p* = 0.20) and 9.38 (95% CI, 1.10 to 80.3; *p* = 0.041), respectively.

**Figure 2:**
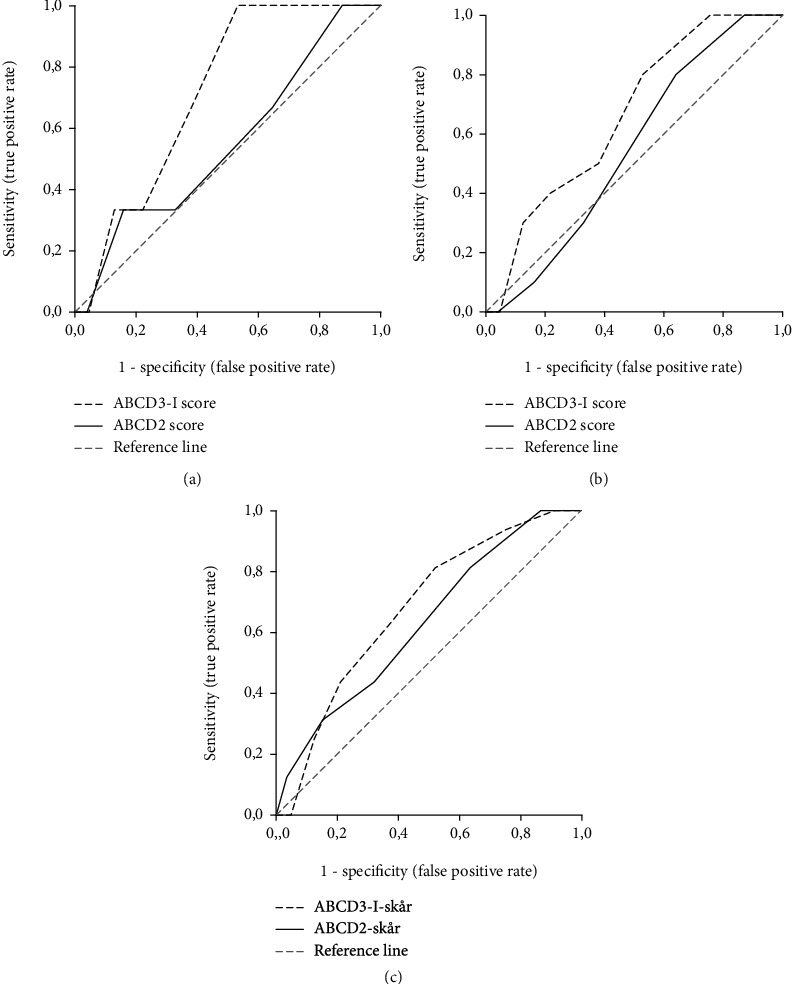
ROC curves of the ABCD3-I score and ABCD2 score at 1 week (a), 3 months (b), and 1 year (c).

The AUC values of ABCD3-I were higher than those of ABCD2 at each time point (see Figure 2), but the difference only reached statistical significance for stroke recurrence at 1 week. AUCs for the ABCD2 score were 0.55 (95% CI, 0.24 to 0.86) within 1 week, 0.55 (95% CI, 0.42 to 0.68) within 3 months, and 0.63 (95% CI, 0.50 to 0.76) within 1 year. AUCs for the ABCD3-I score within the same time points were 0.72 (95% CI, 0.54 to 0.89) (compared with the ABCD2 score, *p* = 0.019), 0.66 (95% CI, 0.53 to 0.80) (*p* = 0.11), and 0.68 (0.95% CI, 0.56 to 0.79) (*p* = 0.39), respectively (see Table 3).

A Cox regression analysis to evaluate the risk of stroke in the presence of positive DWI, dual TIA, and carotid stenosis of the ABCD3-I score compared to none of these characteristics showed hazard ratios of 2.53 (95% CI, 0.95 to 6.73; *p* = 0.064), 1.11 (95% CI, 0.25 to 4.90; *p* = 0.89), and 0.71 (95% CI, 0.09 to 5.39; *p* = 0.74), respectively, for the entire follow-up period of 1 year.

## 4. Discussion

This secondary analysis of the data from the MIDNOR TIA study validated the usefulness of the ABCD3-I score to predict the 1-week, 3-month, and 1-year risk of stroke after TIA. We found an association between the higher ABCD3-I scores and increased stroke risk one week, three months, and one year after TIA both with the use of the AUC values for ABCD3-I and Cox proportional hazards regression analyses comparing the medium- and high-risk with low-risk ABCD3-I score. This is consistent with several previous TIA risk studies that have shown an increase in stroke risk with increasing ABCD3-I score points [12–16]. However, there were few strokes registered and the AUC statistics showed very wide confidence intervals with the lower limit reaching close to 0.5 at every time point in the follow-up period. There were also wide confidence intervals for the hazard ratios reported.

The ABCD2 score was not able to predict stroke after TIA in this cohort with AUC values of 0.55 to 0.63 and the lower limit of the confidence intervals as low as 0.24 within 1 week. Compared to this, the AUC values for the ABCD3-I score were higher, but only significantly for stroke recurrence at 1 week, suggesting that the overall predictive value of the ABCD3-I score is low. We found a very low risk of stroke, and this probably affected the predictive value of the clinical scores in our study. These results are, however, in line with the risks described in our own prospective TIA cohort [7]. Other recent studies reporting the effect of rapid evaluation and treatment initiation of TIA patients have found similar low stroke risks [3, 4, 17, 18]. As described earlier [7], this trend towards a lower stroke recurrence during the recent years may be explained by more rapid evaluation by stroke specialists, better implementation of secondary stroke prevention strategies, and changing risk factors in the population, for instance, through a decline in cigarette smoking rates. The first days to a week after TIA is generally regarded as the time window with the highest stroke risk [19]. In our study, within the first week, only 3 out of 233 patients (1.3%) with an ABCD3 − I score ≥ 4 (moderate to high risk) experienced a stroke. The corresponding numbers for the entire follow-up period of 1 year for the same group were also low—15 out of 233 patients (6.4%). In the low-risk group (score 0-3), there were no registered strokes within 1 week and 3 months, and only 1 stroke within 1 year.

The ABCD3-I score was developed to improve risk scoring accuracy in a specialist setting. It was not intended to be used in the prehospital settings, as DWI (and carotid artery imaging) is generally not available to community-based clinicians who make referrals. Though many studies have pointed out the increased discrimination ability of the ABCD3-I score (compared to the ABCD2 score), there is little evidence on how this score could be implemented in a clinical setting and used in practice. Truly, the clinical context in which a risk score is applied determines its usefulness, and not its predictive power alone. It has been argued that some higher-risk patients could benefit from hospital admission, where they can have immediate access to early acute treatment (thrombolysis and thrombectomy) in case of recurrent strokes [12]. A recent study on the use of the ABCD3-I score in the emergency department reported significantly decreased hospital admissions and cost with similar 90-day neurological outcomes after the initiation of an ABCD3-I-based pathway for TIA evaluation [20]. This was however a small study with statistical methodological limitations, a small sample size, and a short follow-up. It was also based on an emergency department which could perform MRI DWI quickly. The use of DWI is recommended in the investigation of TIA [21, 22]. It is also proposed as the basis for the tissue-based definition of TIA as opposed to the traditional time-based definition, which we used in our study [23]. Our Cox proportional hazards regression on the additional components in the ABCD3-I score supports the relation between positive DWI after TIA and the risk of future strokes, and we agree that such investigation should be done, if available. But the availability of MRI DWI varies greatly between hospitals, regions, and countries, so also in rural districts with small hospitals in a high-income country like Norway.

Interpreting our data, we noticed that patients with a low ABCD2 score and a low ABCD3-I score even more so had an extremely low risk of stroke after TIA. However, due to the generally very low post-TIA stroke risk in our study and in similar contemporary studies [17, 24], both for patients with low and high score, there were no significant differences between the groups. In areas where TIA clinics are not available, one can argue that these scores could be used to identify those low-risk patients who can have assessment beyond the recommended 24-48 hours after TIA [21, 22]. There is strong evidence that early administration of aspirin is a key intervention to prevent stroke after TIA [25]. However, as reasoned for in our primary analysis of the ABCD2 score in our TIA cohort [7], patients with a low score also can have severe underlying pathology; hence, rapid evaluation in a specialized stroke center, either in an outpatient or inpatient setting, seems to be the essential factor for optimizing the outcome in all TIA patients. In our TIA population, almost all patients were admitted immediately to the hospital, underwent rapid TIA assessment (including MRI DWI and extracranial artery investigations), and were medically treated according to guidelines. Consequently, further progression in investigations or treatment did probably not differ greatly between the low- and high-risk groups. This may reduce the usefulness of the ABCD2 and ABCD3-I scores and contribute in explaining why the scores do not discriminate better between the low- and high-risk groups. Atrial fibrillation is a known risk factor for cerebrovascular ischemic events. However, validation studies of post-TIA risk scores have not found an increased predictive stroke risk accuracy by taking atrial fibrillation into account [26, 27]. In our study, when comparing proportions of atrial fibrillation between low- and medium- to high-risk patients, we did not find significant differences. Therefore, adding this to the risk score probably would not have changed our results.

The main strength of our study lies in the prospective methodology collecting a cohort in close collaboration between the local hospitals and the primary health care system. Recruited patients were given early and comprehensive stroke unit care based on current guidelines. This makes it a “real-life” clinical scenario. Additionally, the diagnosis of included patients was made by stroke specialists making inclusion of TIA mimics less likely. We acknowledge that our study has some important limitations. The main limitation is the lack of statistical power due to the low rates of stroke. However, the power calculation in the original study was based on current knowledge of stroke risk after TIA, and the patients included in this analysis had similar stroke rates as the original cohort. Second, the patients that were excluded from the analysis because DWI was not performed or performed too late, or because extracranial imaging was not performed, were older and had generally higher load of vascular risk factors. At the same time, there were several similarities: there were no significant differences in subsequent stroke rates between included and excluded patients. Also, excluded patients had proportions of dual TIAs similar to the included patients (22/272), and patients in this group that did undergo carotid artery imaging had similar rates of carotid stenosis (22/215) as the included patients. Therefore, it is not highly likely that excluding a part of the cohort on the grounds of lack of availability of investigational data would constitute a relevant selection bias. Also important, the baseline clinical characteristics of the included patients were similar to those of comparable TIA stroke prediction studies [12]. Third, the ABCD3-I scores were calculated retrospectively, which could have increased the risk of errors in registration of data. Likewise, the fact that there were few strokes in the follow-up time makes results vulnerable to errors being done in the registration process. In our study, the prevalence of dual TIA was low. The reported prevalence of dual TIA, however, varies widely among different populations in previous studies [6, 14, 16]. As a fact, several of the components of the ABCD2 and ABCD3-I scores are based on patients' own memory, and therefore susceptible to recall bias.

## 5. Conclusions

The ABCD3-I score had limited value in a short-term prediction of subsequent stroke after TIA, and the ability to predict stroke deteriorated further during a long-term follow-up. The ABCD2 score did not predict subsequent stroke accurately at any time point. Due to the low numbers of stroke, the study did not have enough power to detect significant differences in stroke risk between patients with high- and low-risk scores, and our results therefore must be interpreted with caution. They still give an indication that these clinical TIA risk scores are less beneficial to discriminate between the high- and low-stroke-risk groups in populations with a general low risk of stroke after TIA. This is also supported by recent publications and guidelines [21, 28]. We believe that the best approach to TIA patients is to carefully consider each of the components of the investigated scores through rapid assessment and initiation of treatment, rather than using dichotomized scores.

## Figures and Tables

**Table 1:** ABCD2 and ABCD3-I scores.

	ABCD2 score	ABCD3-I score
Age ≥ 60 years	1	1
Blood pressure ≥ 140/90 mmHg	1	1
Clinical features		
Speech impairment without weakness	1	1
Unilateral weakness	2	2
Duration		
10-59 min	1	1
≥60 min	2	2
Diabetes present	1	1
Dual TIA (TIA leading patient to seek medical help plus at least on other TIA in the preceding 7 days)	NA	2
Imaging: ≥50% stenosis of ipsilateral internal carotid artery	NA	2
Imaging: acute MRI-DWI hyperintensity	NA	2
Total range	0-7	0-13

NA: not applicable; TIA: transient ischemic attack; DWI: diffusion-weighted imaging.

**Table 2:** Clinical characteristics in included patients with complete data for analysis of the ABCD2 and ABCD3-I scores and excluded patients, *n* (%) or mean ± SD.

Patient characteristics	Included (*n* = 305)	Excluded (*n* = 272)
Demographics		
Age in years, mean (±SD)	68 (10.9)	73.4 (10.5)
Male	183 (60.0)	144 (52.9)
Age in years, mean ± SD	68.0 ± 10.9	73.4 ± 10.5^∗^
Medical history, *n* (%)		
Former TIA	44 (14.4)	57 (21)
Former ischemic stroke	38 (12.5)	49 (18.0)
Former TIA	44 (14.4)	57 (21.0)^∗^
Former myocardial infarction	33 (10.8)	34 (12.5)
Diabetes mellitus	33 (10.8)	33 (12.1)
Hypertension	140 (45.9)	171 (62.9)^†^^∗^
Hypercholesterolemia	104 (34.1)	112 (41.2)^‡^
Atrial fibrillation	29 (9.5)	50 (18.4)^∗^
Current smoker	55 (18.0)	39 (14.3)
Former smoker	115 (37.7)	107 (39.3)
Modified Rankin score value 0 to1	259 (84.9)	218 (80.1)
ABCD2 score range		
0	4 (1.3)	3 (1.1)
1	5 (1.6)	10 (3.7)
2	29 (9.5)	33 (12.1)
3	70 (23.0)	52 (19.1)
4	97 (31.8)	80 (29.4)
5	51 (16.7)	56 (20.6)
6	37 (12.1)	31 (11.4)
7	12 (3.9)	7 (2.6)
Medication		
At baseline		
Any antiplatelet treatment	113 (37.0)	120 (44.1)
Any anticoagulation	24 (7.9)	32 (11.8)
Blood pressure-lowering agent	140 (45.9)	171 (62.9)^∗^
Lipid-lowering agent	104 (34.1)	112 (41.2)
At discharge		
Any antiplatelet treatment	276 (90.5)	224 (82.4)^∗^
Any anticoagulation	37 (12.1)	54 (19.9)^∗^
Blood pressure-lowering agent	168 (55.1)	188 (69.1)^∗^
Lipid-lowering agent	264 (86.6)	219 (80.5)^∗^
No. of strokes		
<1 week	3 (1.0)	2 (0.7)
<3 months	10 (3.3)	9 (3.3)
<1 year	16 (5.2)	15 (5.5)

^†^Using blood pressure-lowering medication. ^‡^Using lipid-lowering medication. ^∗^Significant difference between the groups (*p* < 0.05).

**Table 3:** The 1-week, 3-month, and 1-year risks of stroke according to cutoff values of the ABCD2 and ABCD3-I scores with corresponding AUC levels.

		Stroke events (% of patients)		
	Patients, *n* (%)	<1 week	<3 months	<1 year
ABCD2 score				
0-3	108 (35.4)	1 (0.9)	2 (1.9)	3 (2.8)
4-7	197 (64.6)	2 (1.0)	8 (4.1)	13 (6.6)
AUC (95% CI)		0.55 (0.24-0.86)	0.55 (0.42-0.68)	0.63 (0.50-0.76)
ABCD3-I score				
0-3	72 (23.6)	0	0	1 (2.1)
4-7	193 (63.3)	2 (1.0)	7 (3.6)	11 (5.7)
8-13	40 (13.1)	1 (2.5)	3 (7.5)	4 (10.0)
AUC (95% CI)		0.72 (0.54-0.89)	0.66 (0.53-0.80)	0.68 (0.56-0.79)
Total no. of strokes	305 (100)	3 (1.0)	10 (3.3)	16 (5.2)

## Data Availability

Deposition of patient level data in a public repository was not specified in the study protocol, which was approved by the ethics committee before the study began. Provided that the Regional Ethics Committee gives approval, patient-level data will be available on request.

## Additional Points

*Statement*. An earlier version of our manuscript was (March 5th, 2020) presented as a preprint (preliminary report) in Research Square.
